# Accuracy of a Wrist-Worn Heart Rate Sensing Device during Elective Pediatric Surgical Procedures

**DOI:** 10.3390/children5030038

**Published:** 2018-03-08

**Authors:** Gloria Pelizzo, Anna Guddo, Aurora Puglisi, Annalisa De Silvestri, Calogero Comparato, Mario Valenza, Emanuele Bordonaro, Valeria Calcaterra

**Affiliations:** 1 Pediatric Surgery Unit, Children’s Hospital, Istituto Mediterraneo di Eccellenza Pediatrica, 90134 Palermo, Italy; emanuelebordonaro@yahoo.it; 2 Anesthesiology and Intensive Care Unit, Children’s Hospital, Istituto Mediterraneo di Eccellenza Pediatrica, 90134 Palermo, Italy; annaguddo@tiscali.it (A.G.); aurora.puglisi@gmail.com (A.P.); 3 Biometry & Clinical Epidemiology, Scientific Direction, Fondazione IRCCS Policlinico San Matteo, 27100 Pavia, Italy; a.desilvestri@smatteo.pv.it; 4 Pediatric Cardiology Unit, Children’s Hospital, Istituto Mediterraneo di Eccellenza Pediatrica, 90134 Palermo, Italy; calogero.comparato@arnascivico.it; 5 Operating Room Coordination, Ospedale ARNAS Civico, Di Cristina e Benfratelli, 90134 Palermo, Italy; mario.valenza@arnascivico.it; 6 Pediatrics and Adolescentology Unit, Department of Internal Medicine, University of Pavia and Fondazione IRCCS Policlinico San Matteo, 27100 Pavia, Italy; v.calcaterra@smatteo.pv.it

**Keywords:** personal fitness tracker, children, pediatric surgery, heart rate

## Abstract

The reliability of wearable photoplethysmography (PPG) sensors to measure heart rate (HR) in hospitalized patients has only been demonstrated in adults. We evaluated the accuracy of HR monitoring with a personal fitness tracker (PFT) in children undergoing surgery. HR monitoring was performed using a wrist-worn PFT (Fitbit Charge HR) in 30 children (8.21 ± 3.09 years) undergoing laparoscopy (*n* = 8) or open surgery (*n* = 22). HR values were analyzed preoperatively and during surgery. The accuracy of HR recordings was compared with measurements recorded during continuous electrocardiographic (cECG) monitoring; HRs derived from continuous monitoring with pulse oximetry (SpO2R) were used as a positive control. PFT-derived HR values were in agreement with those recorded during cECG (*r* = 0.99) and SpO2R (*r* = 0.99) monitoring. PFT performance remained high in children < 8 years (*r* = 0.99), with a weight < 30 kg (*r* = 0.99) and when the HR was < 70 beats per minute (bpm) (*r* = 0.91) or > 140 bpm (*r* = 0.99). PFT accuracy was similar during laparoscopy and open surgery, as well as preoperatively and during the intervention (*r* > 0.9). PFT–derived HR showed excellent accuracy compared with HRs measured by cECG and SpO2R during pediatric surgical procedures. Further clinical evaluation is needed to define whether PFTs can be used in different health care settings.

## 1. Introduction

The growing field of mobile health technologies has created a platform for innovation and new trends in capturing patient health data [1]. Over the last five years, interest has grown regarding the potential use of wearable devices to improve health care delivery [2,3,4,5].

Recently, wearable activity trackers have been developed that use optical blood flow sensing (photoplethysmography, PPG) techniques to measure heart rates (HR) [6]. PPG is a non-invasive method for the detection of HR and optically assesses vascular tissue using a probe, usually light-emitting diode (LED). The PPG sensor probe (e.g., LED lights) shines directly into the skin, evidencing changes in the blood volume to measure HR. HR is determined based on the theory that blood flow through the artery is inversely related to the amount of light refracted. PPG techniques using optical LED blood flow sensors have increased the popularity of novel HR monitoring devices, with many new models entering the market each year [6,7].

In the medical setting, the ability of wearable PPG sensors to reliably measure HR has been extensively documented in the adult age [8,9,10], including hospital in-patients [11] but limited data are available in pediatrics [12,13,14]. We evaluated the accuracy of HR monitoring with a personal fitness tracker (PFT) in hospitalized pediatric patients undergoing elective surgery, in order to evaluate its potential role in pediatric health care. The accuracy of HR recordings was compared with gold standard measurements made with continuous electrocardiographic (cECG) monitoring. 

## 2. Patients and Methods

We used the Fitbit Charge HR (Fitbit, San Francisco, CA, USA) PFT to monitor HRs in 30 patients sequentially admitted to the Pediatric Surgery Unit for minor elective laparoscopic or open surgical procedures. Surgery was performed under general or local anesthesia.

Participants were recruited between 1 February 2017 and 31 May 2017. The eligibility criteria were: (a) 4–16-years of age; (b) males and females (c) no cutaneous anomalies or bone deformity of the arm on which the device was to be placed. Auxological data (weight, height, body mass index and pubertal stage according to Marshall and Tanner [15,16]) were recorded in all patients.

The personal fitness tracker device studied is a wrist-worn device resembling a watch, which uses PPG to detect periodic changes in blood flow beneath the sensor, thereby measuring HR. The study used one PFT (size small), which was assigned a unique email address and log-in credentials on the Fitbit website; for each device an anonymous Fitbit user profile (online “dashboard”) was created. Wristbands were placed on the arm in accordance with the manufacturer’s guidelines.

To test the accuracy and potential utilization in different pediatric surgical settings, HR values were recorded every 5 min for 30 min at two intervals: anesthesia induction (T1) and during surgery (T2) when surgical instruments were used. 

For comparison, HR measurements were recovered from the Intensive Care Unit (ICU) bedside monitors (Infinity Delta, Dräger, Lübeck, Germany). Data included heart rate values recorded during cECG monitoring, as well as heart rate data derived from continuous monitoring with pulse oximetry (SpO2R), as positive controls. We synchronized the bedside monitor data and PFT. 

To assess the potential effects of heart rhythm disorders (although rare in pediatrics) on accuracy, rhythm status was based on examination of cECG recordings both at the time of device application and at device removal, with patients designated as being in sinus rhythm only if this was true at both time points.

### 2.1. Ethical Considerations

The study was performed according to the Declaration of Helsinki and with the approval of the Institutional Review Board of Children’s Hospital, Istituto Mediterraneo di Eccellenza Pediatrica, Palermo, Italy (n.000551 on 31 March 2017). Parents and/or legal guardian, after receiving information about the study, gave their written consent.

The Fitbit Charge HR is a commercially available PFT and is not currently regulated by the US Food and Drug Administration. The study did not receive funding from the device manufacturer or from any other source.

### 2.2. Statistical Analysis

To describe the agreement between continuous measures obtained by different devices, we calculated Lin’s concordance correlation coefficient (CCC). This is expressed as the product of Pearson’s *r* (the measure of precision) and the bias-correction factor (Cb, the measure of accuracy). CCC ranges included values from 0 to +1. Agreement was classified as poor (0.00 to 0.20), fair (0.21 to 0.40), moderate (0.41 to 0.60), good (0.61 to 0.80), or excellent (0.81 to 1.00). The Bland and Altman limits of agreement (LOA), with their 95% confidence interval (CI), within and between observers were reported as well. These represent the interval within which the absolute difference between two repeated test results, even with a high agreement or concordance, may be expected to lie with a probability of 95%. If the differences within means ± 1.96 standard deviation (SD) (LOA) are not clinically important, the two methods may be used interchangeably.

## 3. Results

We enrolled a sample of 30 children (16 male (M)/14 female (F), mean age 8.21 ± 3.09) that met our enrollment criteria. Eight children underwent laparoscopic surgery (3M/5F, mean age 9.8 ± 3.3 years) and 22 underwent traditional open surgery (13M/9F, mean age 7.6 ± 2.7 years). Indications for surgery included abdominal-inguinal pathology (*n* = 18), gynecological mass (*n* = 2), excision of cutaneous–subcutaneous lesions (*n* = 10). The clinical features of the patients are reported in Table 1.

All children were in sinus rhythm. HR means during laparoscopy and open surgery groups were not different (cECG 100.5 ± 21.1 beats per minute (bpm); PFT 100.6 ± 21.2; SpO2R 100.5 ± 21.3, *p* = 0.9).

**Figure 1:**
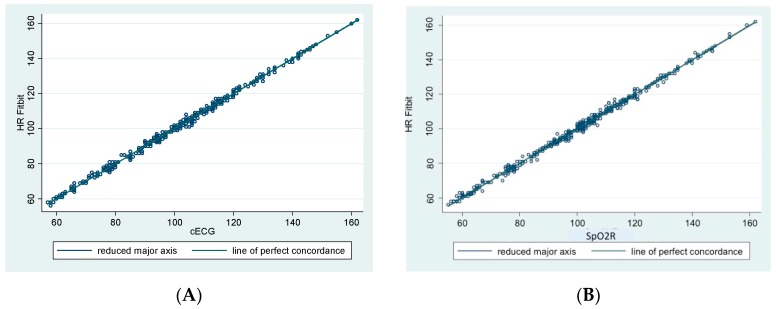
Agreement between personal fitness tracker (PFT) derived heart rate (HR) and HR derived from continuous electrocardiographic monitoring (cECG, Panel **A**) and from continuous monitoring with pulse oximetry method (SpO2R, Panel **B**).

PFT-derived HR values were in agreement with those recorded during cECG monitoring (*r* = 0.99; average bias of −0.05 bpm, 95% CI −2.454–2.43 bpm and SpO2R (*r* = 0.99; average bias of −0.01 bpm, 95% CI −2.8–2.8 bpm), Figure 1.

Agreement in PFT performance remained high in children aged less than 8 years (cECG *r* = 0.99; −0.18 bpm, 95% CI −2.4–2.0; SpO2R *r* = 0.99; −0.13 bpm, 95% CI −2.4–2.1) and in subjects weighing less than 30 kg (cECG *r* = 0.99, −0.12 bpm, 95% CI −2.4–2.1; SpO2R *r* = 0.99, −0.08 bpm, 95% CI −2.8–2.7).

The limits of agreement were similar for the PFT method in comparison with the cECG and SpO2R methods, even when HR was lower than 70 bpm (cECG *r* = 0.95, 0.26 bpm, 95% CI −1.6–2.1; SpO2R *r* = 0.91; 0.54 bpm, 95% CI −2.0–3.1) or higher than 140 bpm (cECG *r* = 0.95, −0.26 bpm, 95% CI −1.6–2.1; SpO2R *r* = 0.99, 0.08 bpm, 95% CI −2.2–2.0). 

PFT accuracy was similar preoperatively (cECG *r* = 0.99; −0.1 bpm, 95% CI −2.7–2.5; SpO2R *r* = 0.99; −0.06 bpm, 95% CI −2.9–3) and during surgery (cECG *r* = 0.99; −0.005 bpm, 95% CI −2.4–2.4; SpO2R *r* = 0.99; −0.04 bpm, 95% CI −2.7–2.6), *p* = 0.4; there were no differences between laparoscopy and traditional open surgery (*p* = 0.2).

## 4. Discussion

In hospitalized pediatric patients undergoing elective surgery, PFT–measured HR showed excellent accuracy in comparison with HR measured during cECG and SpO2R monitoring. Accuracy remained high in children aged less than 8 years and weighing less than 30 kg. These preliminary data will be helpful in defining the potential role of PFT in the pediatric surgical setting.

Advances in wireless technologies and low-power electronics associated with health care are driving innovations in wearable medical devices [17]. Wearable health-related technologies refer to electronic tools that may be worn or inserted onto the body. A main feature of wearable health-related technologies is that they have a hands-free function that enables the user to access his/her own health data while performing daily routine tasks. Other features include accessibility, wearability, comfortability, portability, multi-functionality, usefulness, reliability and practicability [18].

With the inclusion of sophisticated PPG technology, new generation wearable devices, such as Fitbit Charge HR, are useful in monitoring the heart rate and HR-derived algorithms may be used to estimate energy expenditure [19,20,21]. Although many wearable devices for monitoring and tracking physical activity are available to consumers, relatively few research studies have been conducted to determine their efficacy in promoting health and their actual use within clinical populations remains limited. To date, clinical studies have included patients with chronic diseases, such as osteoarthritis, chronic heart failure, diabetes, peripheral neuropathy, or chronic obstructive pulmonary disease [22,23,24,25,26] and have mostly been limited to outpatient and ambulatory settings. More recently, Kroll et al. described the accuracy of PFT heart rate monitoring in adult hospitalized in-patients [11]. 

The ability of wearable FTP to reliably measure HR in the pediatric age has been reported in outpatients with cancer [14], congenital heart disease [12], visual impairment [27] and to promote and/or measure physical activity. At present, no data are available on the role of wearable sensors to monitor HR in hospitalized children. 

In this study, we directly evaluated the accuracy of the HR data and inferred conclusions regarding other concerns such as small wrist size in young patients and possible electrical/electro-magnetic interference by the surgical environment. With a comparison to HRs measured during cECG and SpO2R monitoring, we have shown that Fitbit PFT-derived HR measurements are accurate in young patients undergoing surgery. In children less than 8 years of age, the diameter of the wrist on which the device is to be placed is smaller than wristband size; however, correct positioning of the optical sensor allowed correct measurement of HRs. Moreover, no previous studies have evaluated potential sources of interference caused by surgical instrumentation, such as metal objects with a high iron content that could produce ferromagnetic interference and materials conducting an electric current that could produce their own magnetic field [28]; but in this study, PFT performance remained high during surgery. This result supports the feasibility of wearable technology in a surgical setting. However, more testing of devices under real-life conditions and in different clinical settings are needed to provide objective evidence regarding the accuracy of HR monitoring capabilities among hospitalized children.

Physiological parameters such as HR, blood pressure, and body temperature can provide critical information about the patient’s physical health status. Changes in HR have been shown to predict impending clinical deterioration [29,30,31,32,33]. We chose to test the accuracy of PFT in the operating room in order to make a comparison with data collected by standard measurements and evaluate two different surgical conditions: anesthesia induction and actual operating conditions. In surgical scenarios, wrist-worn heart sensing devices have the potential to enhance inpatient safety by identifying episodes of clinical deterioration during the acute phase immediately after the intervention, faster than current nurse-driven vital signs monitoring practices allow. Additionally, PFT could provide continuous postoperative benefit by providing enhanced HR monitoring and tracking of mobility during convalescence, providing feedback to both the patient and clinician [11].

PFT facilitates the transfer of information without the use of electrical conductors and the transmission of real-time data helps clinicians detect, prevent, and extend care efficiently and ubiquitously. Increased adoption of mobile health technologies should help continuous monitoring of patient progress, identify individuals most in need of prevention and treatment, and streamline patient–doctor communication [34].

We recognize that there are some limitations to this study starting with the relatively small sample size. Secondly, all children in this study were in sinus rhythm and accuracy would differ in patients not in sinus rhythm. Additionally, we synchronized bedside monitored data and PFT, thus HR values were obtained from different devices with separate internal clocks and, consequently, a shorter time interval between the measurements of separate HR cannot be excluded [11]. Finally, the correct positioning of the optical sensor allowed the measurement of HRs in young children in a stable position; however, it is possible that during activity the signal may not be adequate. Therefore, studies using devices appropriate for children are mandatory.

In conclusion, our preliminary findings indicate that wrist-worn devices utilizing PPG are a potentially useful method to monitor HR in hospitalized children. Further clinical studies are needed to confirm the practicality of these wearable trackers in the pediatric health care setting.

## Figures and Tables

**Table 1:** Patients’ clinical data.

	Total (*N* = 30)	Laparoscopy Group (*n* = 8)	Open Surgery Group (*n* = 22)
**Sex (Male/Female)**	16/14	3/5	13/9
**Age (years)**	8.21 ± 3.09	9.8 ± 3.3	7.6 ± 2.7
>8 years	14	4	10
<8 years	16	4	12
**Indication for surgery**			
Abdominal-inguinal pathology	18	6	12
Gynecological ovarian mass	2	2	0
Excision of cutaneous–subcutaneous lesions	10	0	10
**Weight (kg)**	31.3 ± 15.3	43.0 ± 20.1	27.0 ± 10.2
<30 kg (*n*)	16	3	13
>30 kg (*n*)	14	5	9
**Height (cm)**	132.2 ± 23.3	146.0 ± 16.5	118.5 ± 20.9
**Body Mass Index (kg/m^2^)**	20.5 ± 5.0	23.6 ± 5.2	17.0 ± 0.04
**Pubertal Stage**			
Tanner stage 1 (*n*)	22	4	18
Tanner stage 2–3 (*n*)	3	0	3
Tanner stage 4–5 (*n*)	5	4	1
